# Alterations of Oxidative Phosphorylation Complexes in Papillary Thyroid Carcinoma

**DOI:** 10.3390/cells7050040

**Published:** 2018-05-09

**Authors:** Franz A. Zimmermann, Daniel Neureiter, Wolfgang Sperl, Johannes A. Mayr, Barbara Kofler

**Affiliations:** 1 Research Program for Receptor Biochemistry and Tumor Metabolism, University Hospital Salzburg, Paracelsus Medical University, Salzburg 5020, Austria; 2 Department of Pathology, University Hospital Salzburg, Paracelsus Medical University, Salzburg 5020, Austria; d.neureiter@salk.at; 3 Department of Pediatrics, University Hospital Salzburg, Paracelsus Medical University, Salzburg 5020, Austria; w.sperl@salk.at (W.S.); h.mayr@salk.at (J.A.M.)

**Keywords:** papillary thyroid carcinoma, mitochondria, respiratory chain complex I

## Abstract

The papillary thyroid carcinoma (PTC) is the most common malignant tumor of the thyroid gland, with disruptive mutations in mitochondrial complex I subunits reported at very low frequency. Furthermore, metabolic diversity of PTC has been postulated owing to variable messenger RNA (mRNA) expression of genes encoding subunits of the oxidative phosphorylation (OXHPOS) complexes. The aim of the present study was to evaluate the metabolic diversity of the OXPHOS system at the protein level by using immunohistochemical staining. Analysis of 18 human PTCs revealed elevated mitochondrial biogenesis but significantly lower levels of OXPHOS complex I in the tumor tissue (*p* < 0.0001) compared to the adjacent normal tissue. In contrast, OXPHOS complexes II–V were increased in the majority of PTCs. In three PTCs, we found pathologic mutations within mitochondrially encoded complex I subunits. Our data indicate that PTCs are characterized by an oncocytic metabolic signature that is in low complex I is combined with elevated mitochondrial mass and high complex II–V levels, which might be an important factor for tumor formation.

## 1. Introduction

Papillary thyroid carcinoma (PTC) is the most common tumor of the thyroid gland, accounting for 70–80% of all thyroid malignancies. PTC is readily treatable by thyroidectomy and radioiodine therapy, with 92.9% of patients being considered disease-free directly after surgery and among whom there is a very low recurrence rate (1.4% during a median 10.4 years of follow-up) [1]. Women are more frequently affected than men in a ratio ranging from 2:1 to 4:1. Ionizing radiation is the most important and well-documented risk factor for PTC. Usually, PTC shows a papillary structure, covered by cells with eosinophilic cytoplasm and enlarged nuclei [2,3]. A genetic marker of PTC is an activated RET/PTC oncogene, which results from chromosomal rearrangement of the RET tyrosine kinase to the 5′ region of various heterologous genes, appearing in up to 87% of PTCs [4,5]. Also, a mutation in the BRAF gene (BRAF p.V600E) is detected in 45% of PTC samples [4,6]. This mutation is associated with a more aggressive phenotype and reduced expression of iodide-metabolizing genes [7]. Finally, at much lower frequencies, RAS point mutations (16%) and rearrangements of the Neurotrophic Receptor Tyrosine Kinase 1 (NTRK1; 12%) are also found in PTCs [4].

Overall, cancer cells tend to produce their energy by anaerobic glycolysis even in the presence of oxygen, a phenomenon first reported by Otto Warburg [8]. Within the last decade, mitochondria have attracted increasing attention in cancer research, and oxidative phosphorylation (OXPHOS) complex I, especially, was found to play an essential role in distinct cancer types of diverse tissues, including thyroid oncocytic tumors [9,10]. Oncocytomas are tumors characterized by an increase in the amount of mitochondria and are most frequently found in endocrine tissues [11]. The mitochondrial fusion proteins Drp1 and Fis1 are also upregulated in the oncocytic thyroid tumors compared to their non-oncocytic tumors [12]. A massive reduction of OXPHOS complex I was found in oncocytomas by applying immunohistochemical staining and enzymatic analysis [9,10,13]. Sequencing of the mitochondrial DNA (mtDNA) revealed disruptive mutations in 27% to 37% of thyroid oncocytomas, whereas immunohistochemical staining revealed a lack of complex I protein in all oncocytoma tissues examined [9,10,14]. Complex I mutations are more common in tumors with oncocytic features, whereas mutations within complex V subunits are associated with non-oncocytic tumors. Therefore, complex I mutations seem to be very important for the oncocytic phenotype [14]. Complex I deficiency, either isolated or combined with other OXPHOS enzyme deficiencies, can result from defects in mitochondrial (*n* = 7) or nuclear (*n* = 37) DNA-encoded complex I subunits, but also from mutation of mitochondrial transfer RNAs (tRNAs) [15], mutation of assembly factors [16], and defects in mitochondrial transcription and translation [17]. 

Yeh et al. were the first to sequence the mtDNA (covering 25% of the mtDNA genome) in PTCs and found somatic mutations in three of 13 samples [18]. Abu-Amero et al. sequenced the entire mtDNA of 19 human PTCs and detected somatic mtDNA mutations in seven (19%), mostly missense mutations in complex I subunits and, in one case, a frameshift mutation in the ND2 gene [19]. Gasparre et al. reported that in four PTCs, one had a frameshift mutation in the ND3 subunit of complex I and two had possibly damaging mutations in the COXI and ATP6 genes, respectively [10].

As most subunits of the OXPHOS complexes are nuclear encoded, sequencing of mtDNA gives only limited information about the genetic alterations of the OXPHOS complexes in cancer tissues, as shown in thyroid oncocytomas [9]. Furthermore, the consequences of mtDNA and nuclear DNA mutations at the levels of OXPHOS complexes have not been investigated in human PTC samples. Immunohistochemical staining of homogenous tissue samples correlates well with enzymatic analysis, as the OXPHOS system is mainly regulated by the amount of protein [9,10,13]. Therefore, immunohistochemical staining of tissues, especially those with a heterogenous cellular composition, is the method of choice because it accurately reflects the in vivo situation. Several studies have reported that thyroid oncocytoma is characterized by a dramatic loss of complex I and concomitant enhanced mitochondrial biogenesis [9,10], but a detailed analysis of the alterations of the OXPHOS system in PTC is, to our knowledge, missing. Therefore, the aim of the present study was to evaluate mitochondrial biogenesis and the levels of OXPHOS complexes in human PTC specimens.

## 2. Materials and Methods

### 2.1. Patients

Formalin-fixed paraffin-embedded (FFPE) tissue of human thyroid specimens of patients with PTC (*n* = 18) (mean age: 43 years, range: 24–68 years, 78% female) (Table 1) were obtained from the Department of Pathology, Paracelsus Medical University Salzburg. The PTCs were classified according to current TNM staging [20]. The study was performed according to the Austrian Gene Technology Act. Experiments were performed in accordance with the Helsinki declaration of 1975 (revised 1983) and the guidelines of the Salzburg State Ethics Research Committee (ethical agreement: AZ 209-11-E1/823-2006) since it did not include a clinical drug trial or epidemiological investigation. All patients signed an informed consent document concerning the surgical intervention. Furthermore, anonymity of the patients was ensured and the study did not extend to examination of individual case records.

### 2.2. Immunohistochemical Staining and Analysis

Immunohistochemical staining of complex I (subunit NDUFS4, Abcam, Cambridge, UK), complex II (subunit SDHA, Mitosciences, Eugene, OR, USA), complex III (subunit core 2, Mitosciences, Eugene, OR, USA), complex IV (subunit I, Mitosciences, Eugene, OR, USA), complex V (subunit alpha, Mitosciences, Eugene, OR, USA), and porin (31HL, Mitosciences, Eugene, OR, USA) were performed as described previously [13]. Scale bars in the figures correspond to 100 µm.

### 2.3. Mitochondrial DNA Analysis

In eight cases, the tumor area on the FFPE tissue sections was large enough to allow DNA isolation for sequence analysis. DNA extraction and sequencing of the mitochondrially encoded complex I subunits (*ND1*, *ND2*, *ND3*, *ND4*, *ND4L*, *ND5,* and *ND6*) were performed by Sanger sequencing as described previously [9]. The sequence was compared to the human mitochondrial DNA reference sequence, GenBank NC_012920.

### 2.4. Statistical Analysis

We followed the scoring system as described by Zimmermann et al. [21] to quantify differences in the expression levels of OXPHOS complexes and porin between tumor tissue and adjacent normal tissue. In brief, scores were calculated by multiplying the value for the staining intensity (0, no staining; 1, weak staining; 2, moderate staining; 3, strong staining) by the mean percentage of immunopositive cells per high-power field. Quantification was performed independently by two persons, scoring the whole sample of normal and cancer tissue. The mean values are reported here. An “increase” or “decrease” for the tumor tissue was defined as a greater than 25% difference in the score value compared to the corresponding normal tissue. For statistical analysis, Student’s *t*-test was used. The scores obtained for the OXPHOS complexes and porin were compared between cancerous and normal thyroid tissue with a *p*-value of <0.05 for significant differences.

## 3. Results

### 3.1. Enhanced Mitochondrial Biogenesis in PTC

**Figure 1:**
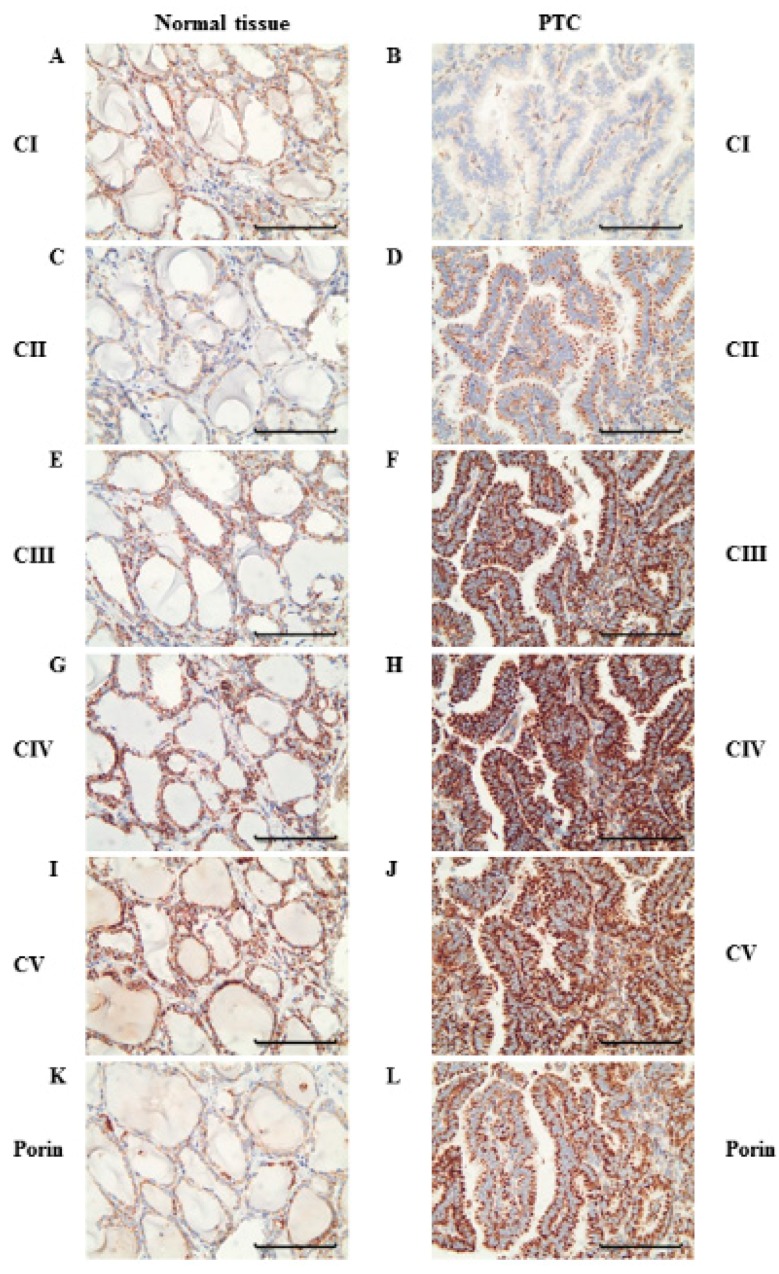
Immunohistochemical staining of the oxidative phosphorylation (OXPHOS) complexes in a representative PTC case (PTC 18). Normal tissue adjacent to PTC 18 shows similar amounts of complex I (**A**), complex II (**C**), complex III (**E**), complex IV (**G**), complex V (**I**), and porin (**K**). PTC 18 shows a reduced amount of complex I (**B**) but increased amounts of complex II (**D**), complex III (**F**), complex IV (**H**), complex V (**J**), and porin (**L**). Scale bar represents 100 µm.

**Figure 2:**
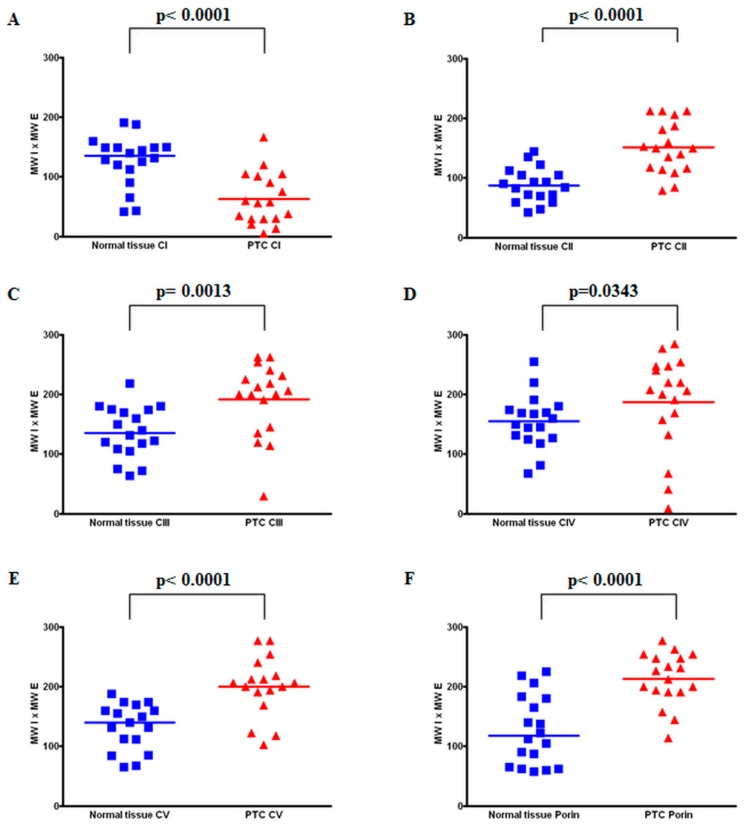
Scoring of all PTC cases. Score values for the staining-intensity of immunopositive cells in normal tissue and PTC tissue (*n* = 18) stained for complex I (**A**), complex II (**B**), complex III (**C**), complex IV (**D**), complex V (**E**), and porin (**F**).

To elucidate potential alterations of mitochondrial biogenesis in PTC compared to normal thyroid tissue, we performed immunohistochemical staining of porin, which is a protein in the outer mitochondrial membrane that is frequently used as a marker for mitochondrial mass [22]. In 14 of 18 PTCs, the level of porin staining was at least 25% higher compared to that of the adjacent normal tissue (average increase: 198%; range 104–363%; *p* < 0.0001) (Figure 1 and Figure 2, Table 1). In four tissues, the level of porin staining in PTCs was similar to that of adjacent normal tissue.

### 3.2. Levels of OXPHOS Complexes in PTC Reveal an Oncocytic Signature

**Figure 3:**
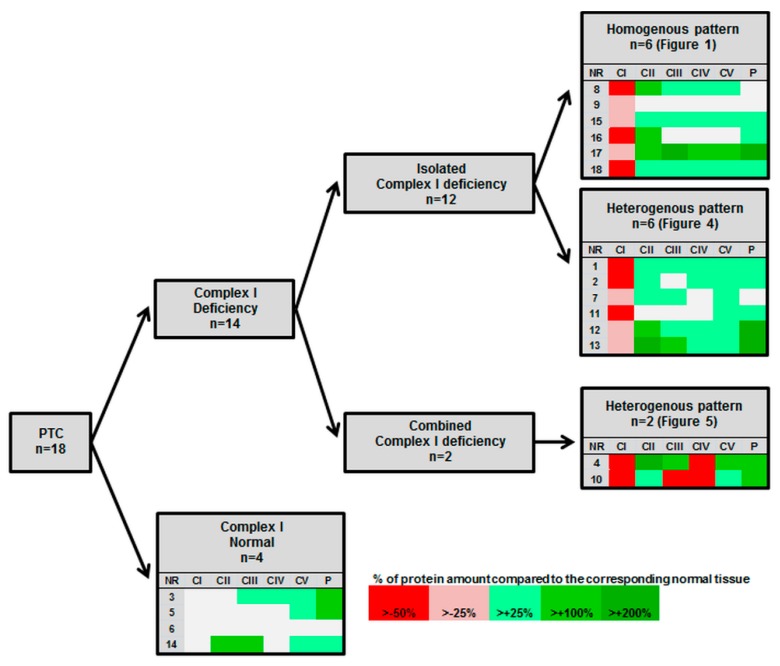
Overview of alterations of OXPHOS complexes in the 18 studied PTCs.

To elucidate the metabolic signature of the aerobic energy metabolism of PTCs, we next examined the expression of OXPHOS complexes by immunohistochemical staining. In 14 of 18 PTCs, lower levels (<75% of the normal tissue) of OXPHOS complex I was detected in the tumor tissue compared to the corresponding normal tissue (average decrease in all 18 PTCs: 51%; range 10–104%; *p* < 0.0001) (Figure 1, Figure 2 and Figure 3, Table 1).

**Figure 4:**
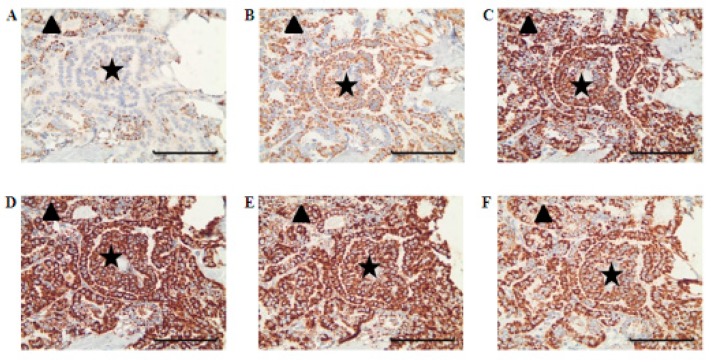
Immunohistochemical staining of OXPHOS complexes in a case with heterogenous deficiency. PTC 13 stained with complex I (**A**), complex II (**B**), complex III (**C**), complex IV (**D**), complex V (**E**), and porin (**F**) antibodies. Areas staining positive for complex I are marked with a triangle, and areas staining negative for complex I are marked with a star. Scale bar represents 100 µm.

In the four PTCs without complex I reduction, the complex I score values in the tumor sections were within the range of the corresponding normal tissue. Among the 14 cases with low complex I staining, 12 showed an exclusive decrease of complex I (Figure 1, Figure 2 and Figure 3, Table 1). Half exhibited a homogenous pattern of low complex I staining (Figure 1), and the other half showed a heterogenous complex I staining pattern (Figure 4).

**Figure 5:**
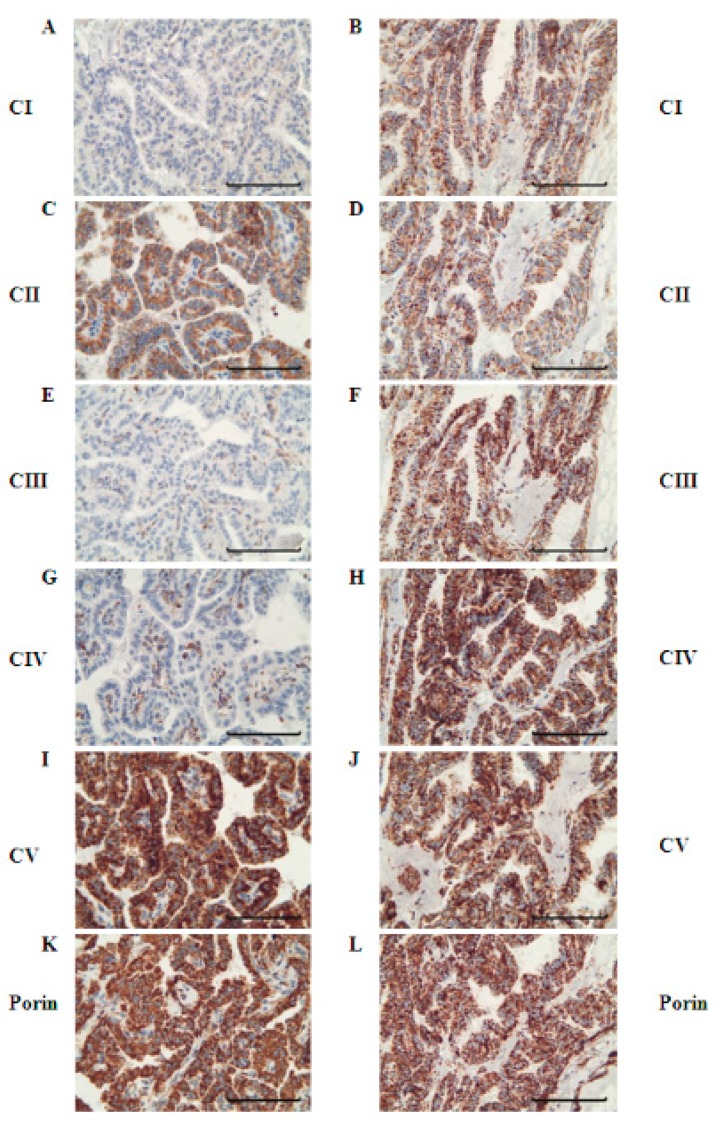
Immunohistochemical staining of OXPHOS complexes in a case with combined OXPHOS deficiency. PTC 10 stained for complex I (**A**,**B**), complex II (**C**,**D**), complex III (**E**,**F**), complex IV (**G**,**H**), complex V (**I**,**J**), and porin (**K**,**L**). Areas within one tumor sample stained negative for complex I (**A**) + III (**E**) + IV (**G**), but some areas stained positive for complex I (**B**) + III (**F**) + IV (**H**). The whole tumor stained positive for complex II (**C**,**D**), complex V (**I**,**J**), and porin (**K**,**L**). Scale bar represents 100 µm.

In two cases with reduced complex I, other OXPHOS complexes were also reduced. PTC 4 showed a combined complex I and complex IV deficiency and PTC 10 showed a combined complex I, III, and IV deficiency. Interestingly, the areas with the combined deficiencies were heterogenous in their OXPHOS-complex expression patterns, in that they were side by side with cancer cell clusters with normal levels of these complexes (Figure 5).

Apart from these two cases with combined OXPHOS deficiency, the levels of staining of complexes II–V were mostly higher compared to that of the corresponding normal tissue (Figure 1 and Figure 2, Table 1). The percentages of PTCs with elevated expression of OXPHOS complexes were 72% for complex II (average increase: 189%, range: 84–353%), 66% for complex III (average increase: 151%, range: 25–234%), 50% for complex IV (average increase: 119%, range: 13–191%), and 88% for complex V (average increase: 158%, range: 92–296%). PTC 6 was the only specimen for which no differences in the levels of OXPHOS complexes and porin were detected. 

Mitochondrial mass (porin staining) was not correlated with the degree of alterations of the OXPHOS complexes (Table 1).

### 3.3. mtDNA Analysis

To elucidate whether mutation of the mtDNA-encoded subunits could explain complex I deficiency in the PTCs, we performed sequence analysis of mtDNA-encoded complex I subunits. The tumor area of eight PTC samples was large enough to allow dissection and sequence analysis. In three PTCs, potentially pathologic mutations within mitochondrially encoded complex I subunits were found. PTC 7 had a 4611_4612delA (MT-ND2) mutation, PTC 8 showed a 11179_11180insT (MT-ND4) mutation and PTC 1 harbored a 14451_14452insT (MT-ND6) mutation. All three mutations were not described previously in the MitoMap database, but are within the first two-thirds of the coding sequence and are expected to cause translational frameshifts (Supplemental Figure S1). 

## 4. Discussion

Above, we described a significant reduction of OXPHOS complex I in PTCs, whereas other enzymes of the OXPHOS system were upregulated in most cases. In three of the cases, a causative defect was found by the identification of disruptive frameshift mutations in mitochondrially encoded complex I subunits. Such frameshift mutations were also described for oncocytomas of the thyroid in a previous study [9]. In thyroid oncocytomas, potentially pathogenic mtDNA mutations were identified in 56% of the analyzed samples [9,10,14], whereas in the present study, loss-of-function mutations in complex I subunits were found in 38% (3/8) of the investigated samples. 

Complex I mutations have been described several times in different types of human cancer, e.g., liver cancer [23], colorectal carcinoma [24], and prostate cancer [25], but at very low frequencies. However, complex I mutations have been found in half of all oncocytomas, such that complex I is regarded as a mitochondrial tumor suppressor in oncocytic tumors [13]. Complex I in thyroid oncocytomas showed a residual protein amount of 4% of the corresponding normal tissue, compared to 51% residual protein in PTCs. In contrast, the level of complex V was increased in both thyroid oncocytomas (149% of the corresponding normal tissue) and PTCs (158%) to nearly the same extent. Although the reduction of complex I in PTCs is less pronounced than in oncocytomas, the majority of PTCs are characterized by an oncocytic metabolic signature. According to a recent study by Cavadas et al. the oncocytic phenotype is secondary to PTC, mostly driven by the accumulation of mitochondrial DNA mutations [26]. This is in accordance with our findings demonstrating a higher degree of complex I reduction in oncocytomas compared to PTC. Mutations of mitochondrial complex I genes in PTCs have been reported in the medical literature, but only in a very low percentage (5–9%) of analyzed cases [10,19]. In our study, we found disruptive complex I mutations in 38% of the analyzed samples. The prevalence of mutations in our study might have been even higher if we had not excluded samples from mtDNA sequencing analysis that demonstrated no reduction of complex I in immunohistochemical staining, since complex I could still be affected by pathogenic mutations other than those leading to protein degradation and disruption of complex I assembly [27].

In four samples, we found no reduction in the amount of complex I. In these samples, it is possible that the enzymatic activity of complex I might still be affected, but not the amount of the enzyme. Measurement of enzymatic activity was not possible in this study because we had only FFPE tissue available. However, our findings of low complex I levels in PTC are in line with the functional analysis of two thyroid tumor cell lines which revealed a severe defect in complex I activity [19]. In another study using gene set enrichment analysis of 105 PTCs, Lee et al. reported that the expression of OXPHOS genes was significantly lower in primary thyroid cancer compared to matched normal tissue [28]. A more in-depth analysis of a subset of the data revealed that only 16% of the PTCs did not show a reduction of OXPHOS RNAs, indicating metabolic diversity, which we also observed in our study but not with the same metabolic pattern.

The same authors additionally presented results of immunohistochemical staining of the complex I subunit NDUFA5 of PTCs (data from Human Protein Atlas), which showed heterogenous areas of lower and similar staining intensities compared to that of the adjacent normal tissue [28]. The heterogenous areas of lower complex I levels are in accordance with our findings. As messenger RNA (mRNA) expression levels do not necessarily correlate with protein levels, it is unfortunate that Lee et al. did not present the expression of all OXPHOS complexes at the protein level. Additionally, we evaluated immunohistochemical staining of different OXPHOS subunits in PTCs available at the Human Protein Atlas (http://www.proteinatlas.org/) and found moderate to strong expression of OXPHOS complexes and porin, which is in accordance with our study results.

At least four subunits (NDUFS1, NDUFS2, NDUFS3, NDUFV1) of complex I have been found to play an important role in both caspase-dependent and caspase-independent apoptotic pathways [29,30,31]. These subunits are cleaved by either granzyme A or granzyme B, which are released by cytotoxic T lymphocytes and natural killer cells. Cleavage of these subunits results in the generation of reactive oxygen species and triggers apoptosis by a caspase-independent pathway. Additionally, NDUFS1 is also cleaved by caspase-3, thereby promoting caspase-dependent apoptosis [29,30,31]. Cancer cells with a reduced amount or absence of complex I might, therefore, gain a substantial survival advantage compared to complex I positive cancer cells. A compensatory increase of the other OXPHOS complexes might attenuate the complex I deficiency in respect to the metabolic function, but they might not be able to compensate for the function of complex I in apoptosis.

## 5. Conclusions

The data we presented in this study show that in the majority of PTCs, OXPHOS complex I is significantly reduced, accompanied by elevation of mitochondrial biogenesis and complexes II–V, which is a similar feature of oncocytic tumors, which are usually benign. The malignant PTCs showed a more-patchy reduction of complex I. In the present study, we found complex I mutations in 38% of the investigated PTC samples, which confirms that the protein reduction is due to a genetic defect. Complex I deficiency might help cells during tumor development to escape different apoptotic pathways.

## Figures and Tables

**Table 1:** Patient data, immunohistochemical scoring, and mutation analysis of papillary thyroid carcinoma (PTC) tissue.

Nr	Age	Sex	CI	CII	CIII	CIV	CV	P	Mutation of Complex I Genes
1	34	f	22	198	157	176	125	130	m. 14451_14452insT
2	45	f	34	161	118	130	199	180	n.d.
3	58	f	88	117	159	160	145	251	n.d.
4	24	m	12	316	228	13	296	235	none
5	27	m	91	104	115	97	180	292	n.d.
6	27	m	104	86	91	109	102	110	n.d.
7	38	f	53	173	137	94	129	123	m. 4611delA
8	64	f	16	202	160	171	125	116	m. 11179_11180insT
9	30	f	58	84	106	104	92	104	n.d.
10	48	f	10	220	25	31	183	233	none
11	60	m	17	97	108	83	143	158	none
12	32	f	53	229	152	138	162	320	n.d.
13	35	f	71	353	214	191	163	333	n.d.
14	36	f	86	209	234	100	170	150	n.d.
15	46	f	73	126	132	138	133	130	n.d.
16	53	f	23	258	115	94	-	168	none
17	68	f	61	285	313	146	183	363	n.d.
18	41	f	38	181	146	168	159	165	none

Complex I (CI), complex II (CII), complex III (CIII), complex IV (CIV), complex V (CV), porin (P), n.d., no mitochondrial DNA (mtDNA) sequence analysis was performed; m, male; f, female.

## Supplementary Materials

The following are available online at www.mdpi.com/2073-4409/7/5/40/s1. Figure S1: Sequencing analysis of the mtDNA of PTC 7 (A) (m.4611delA), PTC 8 (B) (m.11179_11180insT) and PTC 1 (C) (m.14451_14452insT).
