# Supplementary material for: Alterations of Oxidative Phosphorylation Complexes in Papillary Thyroid Carcinoma
Source: Cells. 2018 May 9;7(5):40. doi: 10.3390/cells7050040 (PMC5981264; doi:10.3390/cells7050040)
Supplement: Supplementary file 1 [file cells-07-00040-s001.pdf]

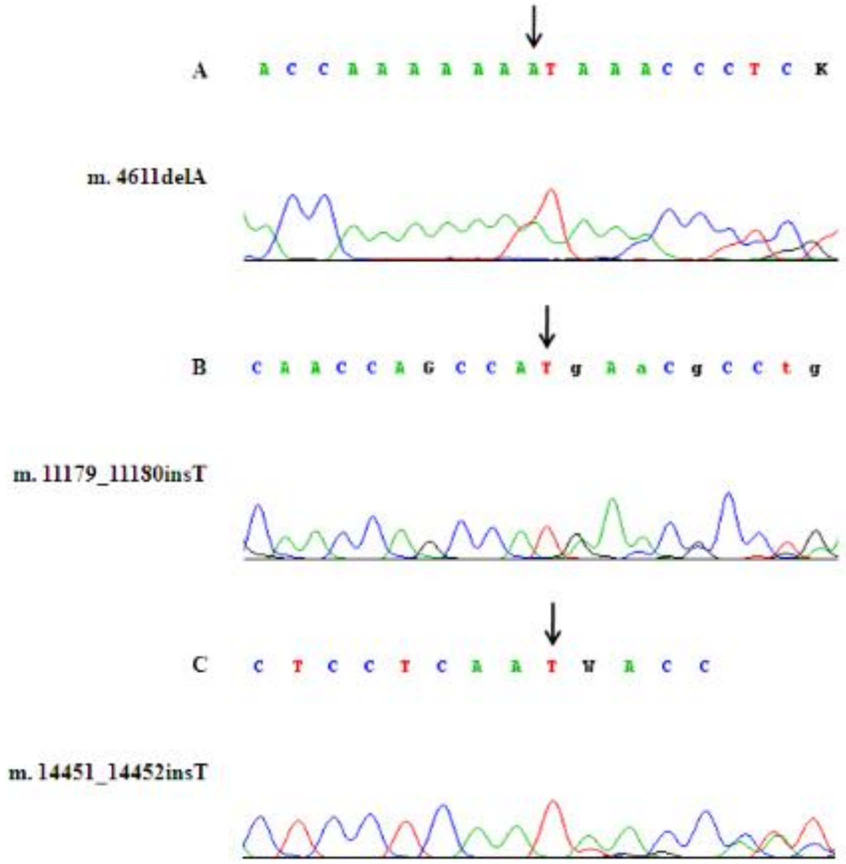

SUPPLEMENTAL FIGURE 1

Figure S1. Sequencing analysis of the mtDNA of PTC 7 (A) (m.4611delA), PTC 8 (B) (m.11179_11180insT) and PTC 1 (C) (m.14451_14452insT).
